# Enhanced Field Emission Properties of Au/SnSe Nano-heterostructure: A Combined Experimental and Theoretical Investigation

**DOI:** 10.1038/s41598-020-58840-8

**Published:** 2020-02-11

**Authors:** Sachin R. Rondiya, Chandradip D. Jadhav, Padmakar G. Chavan, Nelson Y. Dzade

**Affiliations:** 1 The School of Chemistry, Cardiff University, Cardiff, CF10 3AT Wales UK; 2 Department of Physics, School of Physical Sciences, Kavayitri Bahinabai Chaudhari North Maharashtra University, Jalgaon, 425001 India

**Keywords:** Electronic devices, Electronic structure

## Abstract

We report the field emission properties of two-dimensional SnSe nanosheets (NSs) and Au/SnSe nano-heterostructure (NHS) prepared by a simple and economical route of one-pot colloidal and sputtering technique. Field Emission Scanning Electron Microscope (FESEM) analysis reveal surface protrusions and morphology modification of the SnSe NSs by Au deposition. By decorating the SnSe NSs with Au nanoparticles, significant improvement in field emission characteristics were observed. A significant reduction in the turn-on field from 2.25 V/µm for the SnSe NSs to 1.25 V/µm for the Au/SnSe NHS was observed. Emission current density of 300 µA/cm^2^ has been achieved at an applied field of 4.00 and 1.91 V/µm for SnSe NSs and Au/SnSe NHS, respectively. Analysis of the emission current as a function of time also demonstrated the robustness of the present Au/SnSe NHS. Consistent with the experimental data, our complementary first-principles DFT calculations predict lower work function for the Au/SnSe NHS compared to the SnSe NSs as the primary origin for improved field emission. The present study has evidently provided a rational heterostructure strategy for improving various field emission related applications via surface and electronic modifications of the nanostructures.

## Introduction

Nanostructures and nanomaterials have attracted research interest in recent years owing to their demonstrated unique and superior properties over their conventional bulk materials^1,2^. Due to their tunable optoelectronic, magnetic and chemical properties, nanostructured materials have been explored for potential applications in energy generation, optoelectronics, catalysis, and biomedicine. The chemical versatility and unique geometric features of 1-Dimensional (1D) nanostructures make them attractive materials for applications in photoelectronic devices, sensors, energy harvesting, storage, and conversion^3–8^. Recently, 2-Dimensional (2D) nanomaterials have also been attracting significant attention due to their suitable physical and electronic properties for a variety of applications. 2D nanostructures have high density of charge carriers that can easily be transported via a network of lattices and across thousands of interatomic distances with minimal scattering^9^. Owing to their superior charge carriers transport characteristics, 2D nanostructures have received widespread use in optoelectronics, heterogeneous catalysis, energy generation and storage, composites for sensors, field emission, and solar cells applications^10–12^. For field emission applications, a variety of 2D nanostructured including but not limited to graphene, reduced graphene oxide (RGO), MoS_2_, WS_2_ and SnSe been synthesized and explored. Field emission is a versatile technique for generating electron beams, where the electrons are extracted from the surface of a metal/semiconductor by an electrostatic field through quantum mechanical tunneling. Electron emission from the surface of materials is not only limited to fundamental study but it has tremendous applications in the area of telecommunication satellites, medical devices, space research, X-ray sources and electronic displays^13^. For the aforesaid applications to be realized, the emitter material should deliver superior field emission characteristics such as reduced turn-on field, high field emission current density and stability. Amongst all, the turn-on field is one of the most important parameters for practical application purposes. In the last few decades, several strategies including doping with foreign atoms and heterostructure formation have been explored in attempt to improve the turn-on field of emitter materials^14,15^. Compared to the doping approach, heterostructure designs have shown great promise for improving the turn-on field. Zhao *et al*.^16^ reported enhancement in field emission properties of Si nanowires after Au nanoparticle decoration. Drastic improvement in the current density of ZnO nanopillers has been achieved by Chang *et al*.^17^ through surface modification with gold nanoparticles. In recent studies, Patil *et al*. has also showed tuning of the turn-on field for Au/TiO_2_ nanotubes^18^ as well as for Ag/TiO_2_ nanotubes^19^.

Tin selenide (SnSe), an inorganic layered metal chalcogenide material with direct and indirect band gap of 0.9 eV and 1.3 eV, respectively, is a promising material for field emission applications. Because of its layered 2D structure and suitable optoelectronic properties, SnSe is considered an attractive potential candidate for photovoltaic^20,21^ and optoelectronic^22,23^ applications. Single crystals of SnSe have also been explored for thermoelectric applications and found to exhibit high figure of merit (ZT) value of 2.6^24,25^. Very recently, a significantly enhanced field emission characteristics was demonstrated for SnSe nanoflowers through structural and surface morphologies modifications^26^. In the present work, atomically smooth NSs of SnSe have been synthesized chemically and the field emission properties of the SnSe NSs and Au/SnSe nano-heterostructures was systematically characterized.

Field emission efficiency is strongly dependent on different properties of emitter material including but not limited to the work function, electrical conductivity, emitter geometry, emitter alignment, emitter density, and the emitter adhesion to substrates. The work function is the material property that dictates the electron emission capability. Hence, the precise determination of the work function of the emitter is crucial for the rational design of effective field emission materials. Density functional calculations have become indispensable in the rational design of hybrid heterostructures as they are capable of unraveling surface and interface phenomenon, predicting stable heterostructure geometries and their electronic properties. Herein, we present a simple and yet very effective method for synthesizing the SnSe NSs and Au/SnSe NHS with superior field emission performance. Surface modification of the SnSe NSs via Au nanoparticles decoration, is demonstrated to significantly enhance the field emission characteristics of the resulting Au/SnSe NHS. Ultra-low turn-on field and reliable high emission current density was obtained through the Au/SnSe nanocomposite formation. Through first-principles Density Functional Theory (DFT) calculations. We have provided atomic-level insights into the structure of the Au/SnSe NHS and the corresponding work function tuning. Compared to previous reports, the novelty and state-of-the-art of the present study are centred on the synergy between computation and experiment to rationally design Au/SnSe nanocomposite and to derive (sub)atomic-level insights into structure-property relationships for efficient field emission applications.

## Results and Discussions

**Figure 1:**
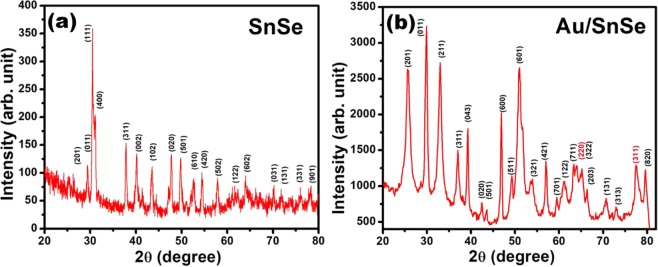
X-ray diffraction patterns of SnSe NSs and Au/SnSe NHS.

**Figure 2:**
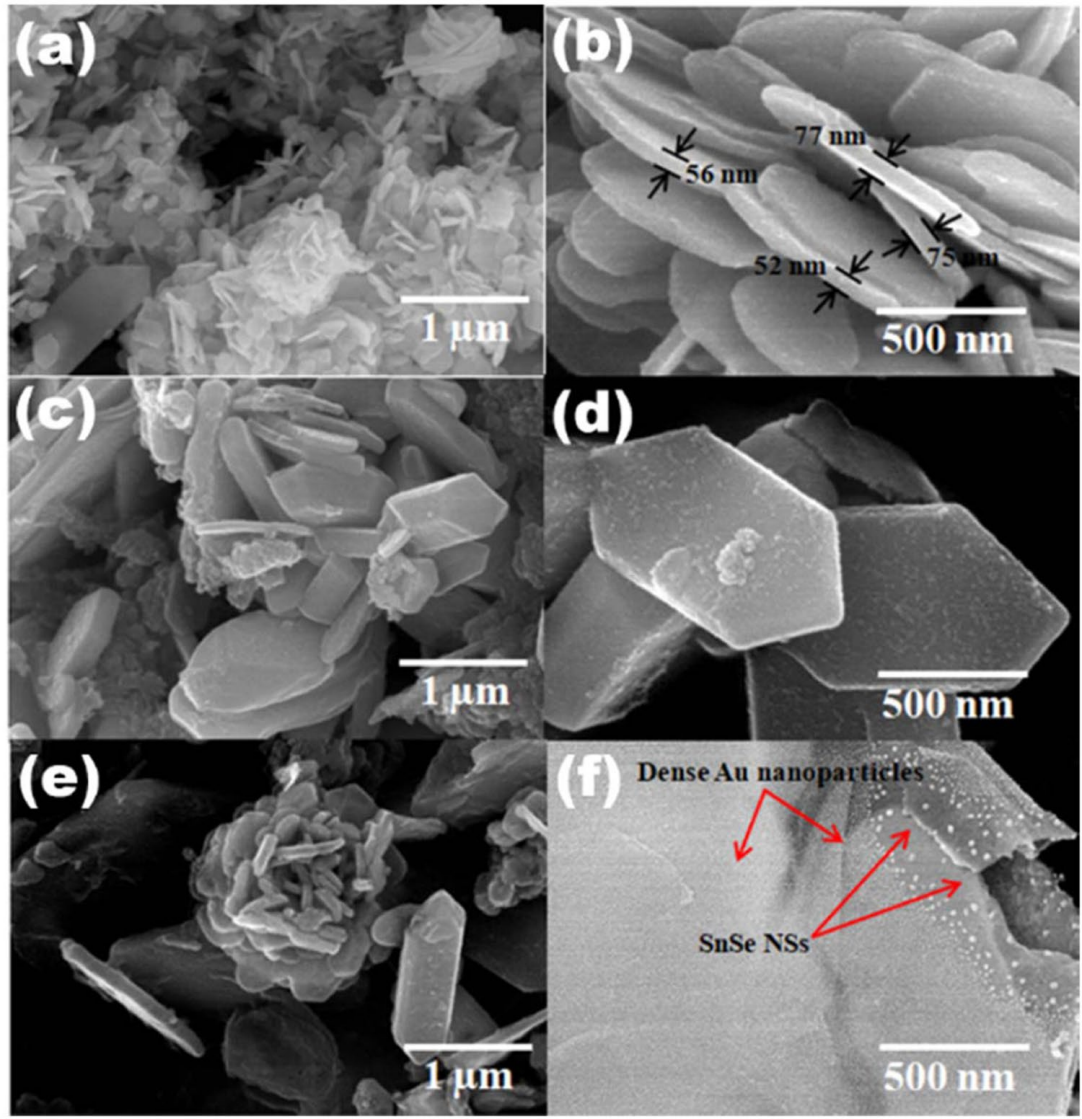
FESEM images of SnSe NSs, Au coated SnSe NSs and Au/SnSe NHS recorded at different magnifications.

The XRD patterns of as-synthesized SnSe NSs and Au/SnSe NHS are shown in Fig. 1. All observed diffraction peaks of SnSe NSs could be index to orthorhombic SnSe with lattice parameters of a = 11.50, b = 4.153 and c = 4.450 Å (JCPDS no. 89-0232). In the case of Au/SnSe NHS (Fig. 1(b)) all diffraction peaks except 64.1, 77.4 and 39.3 are index to orthorhombic SnSe with cell parameters a = 11.16, b = 4.255 and c = 4.358 Å (JCPDS no. 89-0241). The peaks at 64.1 and 77.4 are due to cubic Au^27^ with lattice parameters a = 4.078 Å (JCPDS no. 04-0784). The presence of narrow peak of Se at 39.3 indicates the limited reactivity of Se during reaction. The clear difference observed in the diffraction patterns between the SnSe NSs and Au/SnSe NHs can be rationalized considering the fact that the pattern for the Au/SnSe represents the combination of two sets of patterns: one from porous SnSe NSs and other derives from the Au nanoparticles. Thus, the formation of the Au/SnSe NHs is confirmed from the XRD spectra. Consistent with this, the Field Emission Scanning Electron Microscope (FESEM) images reveal the decoration of tiny Au particles (average diameter 15 nm) on the entire surface of SnSe NSs to form the Au/SnSe NHS are shown in Fig. 2. The absence of characteristic (111) peak Au/SnSe NHS could be attributed the effect of annealing temperature on film texture as has been observed in previous works^28,29^. Low magnification image shown in Fig. 2(a) depicts large coverage of the SnSe NSs. The average thickness of SnSe NSs is found to be 64 nm (Fig. 2(b)). A careful observation indicates thin layer of Au coating on SnSe NSs as shown in Fig. 2(c,d). Annealing process resulted in the thermal breakdown of Au thin film leading to the formation of Au nanoparticles as shown in Fig. 2(e,f). High magnification image (Fig. 2(f)) depicts dense Au nanoparticle coverage on the SnSe NSs, indicating the formation of Au/SnSe NHS.

**Figure 3:**
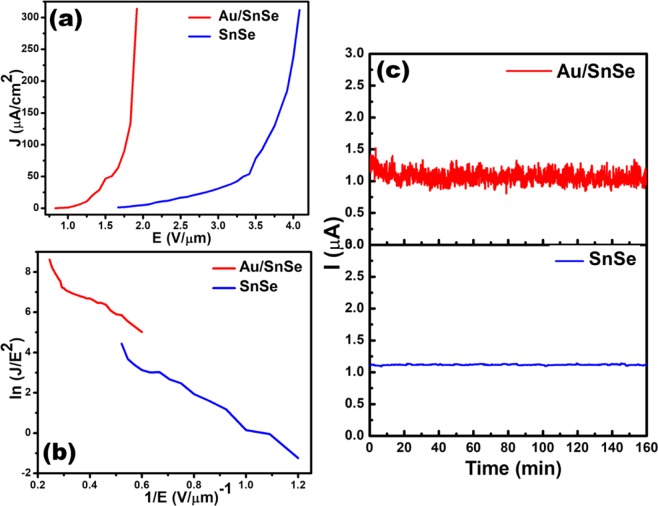
(**a**) J-E and (**b**) F-N plots of SnSe NSs and Au/SnSe NHS (**c**) I–t plot of SnSe NSs and Au/SnSe NHS recorded for 1 µA preset value.

**Figure 4:**
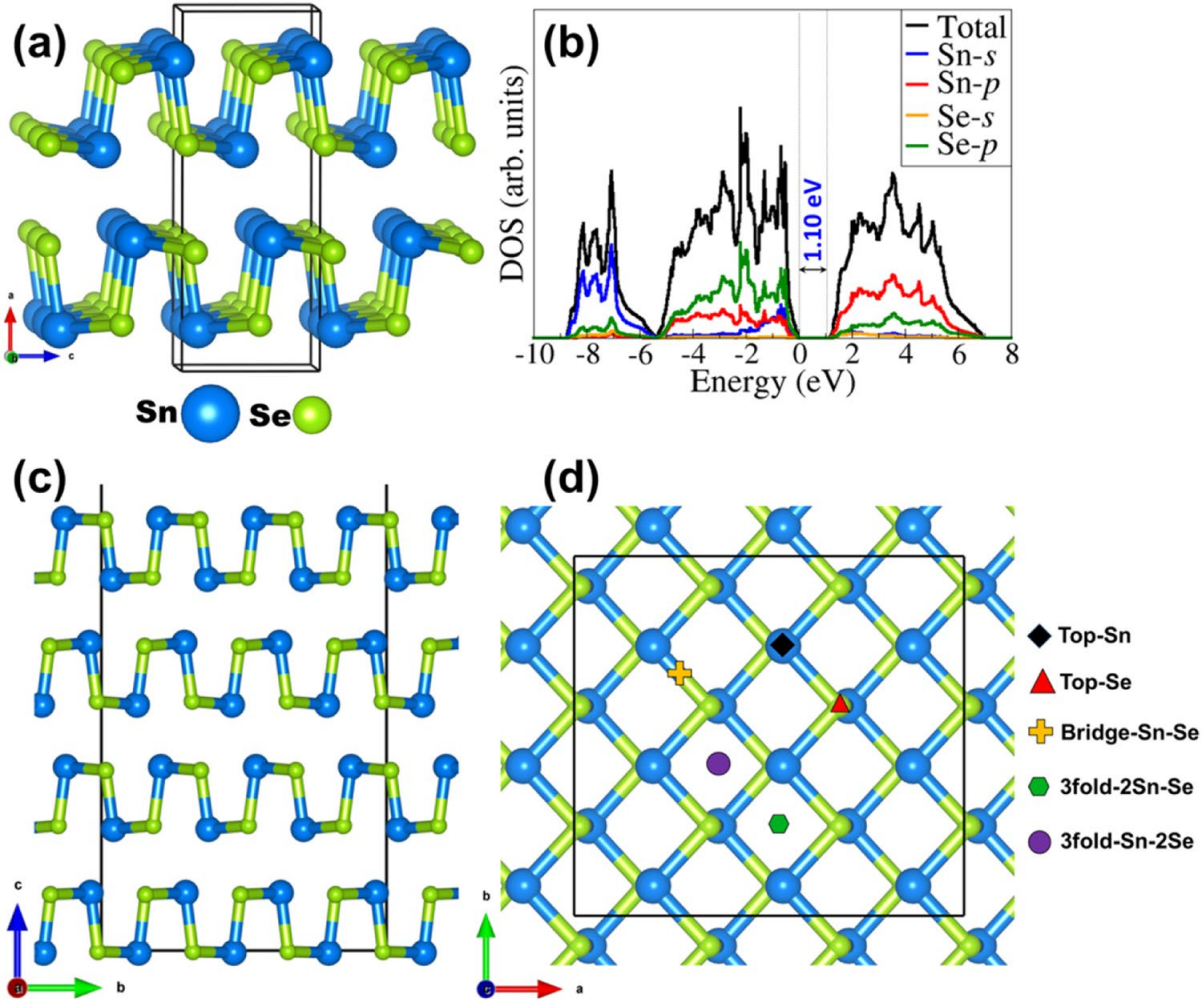
(**a**) Crystal structure and (**b**) the partial density of states (PDOS) of bulk SnSe. The SnSe (100) surface in the side (**c**) and top (**d**) views showing the different Cu adsorption sites explored.

**Figure 5:**
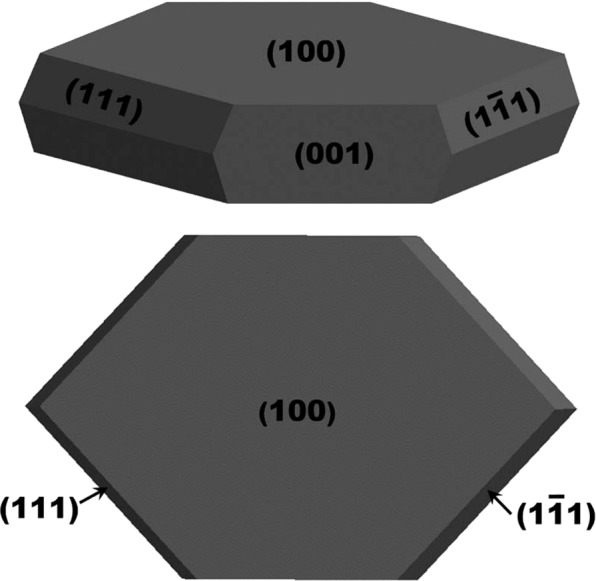
Simulated equilibrium Wulff shape of SnSe nanocrystal.

The field emission current versus applied field (J-E) characteristic of the SnSe NSs and Au/SnSe NHS is shown in Fig. 3(a). In this study, the field required to draw emission current density of 10 µA/cm^2^ is defined as the turn-on field. A drastic reduction in the turn-on field from 2.25 V/µm for the SnSe NSs to 1.25 V/µm for the Au/SnSe NHS was observed. The observed turn-on field of the Au/SnSe NHS is found to be superior when compared with previously investigated semiconducting chalcogenides and NHS as summarized in Table 1 ^16–18,30–35^. The enhanced field emission characteristics can be ascribed to the surface modification of Au/SnSe NHS as evident in the FESEM analysis (Fig. 2(f)), showing the formation of highly dense Au nanoparticles on the entire surface of the SnSe NSs. High local electric field is induced across the emitter surface decorated with the Au nanoparticles (average diameter 15 nm). Generally, surfaces with high nano-protrusions are known to be ‘best’ for field emissionr applications^12^. The maximum current density of 300 µA/cm^2^ was attained for the SnSe NSs and Au/SnSe NHS at an applied field of 4 and 1.9 V/µm, respectively. Consistent with the semiconducting nature of the SnSe NSs^36^, the Fowler-Nordheim (F-N) plots of the SnSe NSs and the Au/SnSe NHS shown in Fig. 3(b) exhibits a non-linear characteristic. Besides the increased performance, the stability of the field emission current is an important parameter for device fabrication considerations. Shown in Fig. 3(c) is the emission current versus time (I-t) plot of the SnSe NSs and Au/SnSe NHS. Preset value of emission current of 1 µA has been chosen for the investigation of the I-t plot for time duration of more than 2 h. Over a continuous of 2 h period of testing, a quite stable emission current was recorded for both SnSe NSs and Au/SnSe NHS. Compared to the SnSe NSs, we observed slightly higher instabilities in the Au/SnSe NHS, which can be attributed to its high surface area that favors higher adsorption of residual gas molecules^14^. Considering that field emission is a surface sensitive phenomenon, any adsorption/desorption over emitter surface directly affects the surface properties and hence the electron emission mechanism^37^. Hence the observed fluctuations in the field emission current can be attributed to residual gas molecules over the emitter surface. To gain an atomic-level insight into the work function tuning of SnSe by adsorbed Au clusters, we have performed first-principles DFT calculations to predict the band gap of bulk SnSe. The optimized crystal structure of bulk SnSe and the corresponding electronic projected density of state (PDOS) are shown in Fig. 4(a,b). The bandgap is predicted at 1.10 eV in close agreement with experiment. It is evident from the PDOS plot that the top of the valence band is dominated by the Se-*p* with contributions from the *s* and *p* states of Sn. The conduction band edge on the other hand is composed mainly of Sn-*p* states, with small contribution from the *s* and *p* states of Se. Next, we investigated the effect of Au deposition on the work function of the SnSe nanosheets. To determine the facet to be employed in the characterization of the Au/SnSe nanocomposite, we have first cut and characterized the surface stabilities of the (100), (001), (011), and (111) SnSe surfaces and their expression in the equilibrium crystal morphology based on Wulff construction. The surface energy of the (100), (001), (011), and (111) surfaces is calculated at 0.14, 0.37, 0.67, and 0.48 Jm^−2^, respectively. The (100) surface is by far the most stable surface of SnSe because its creation only involves breaking the weak vdW interactions between the SnSe layers. The higher surface energies for the (001), (011), and (111) surfaces on the other hand reflect the fact that their creation requires breaking of the most Sn–Se bonds. Consistent with its relative stability, the (100) facet is the most highly expressed facet in the Wulff constructed equilibrium morphology of SnSe as shown in Fig. 5. The predicted thin tabular crystals with the basal (100) facet and the edges composed of the (001) and (111) facets is in excellent agreement with the experimental field emission scanning electron microscope (FESEM) results (Fig. 2). The nonexistence of the (011) surface reflection calculated morphology of the SnSe crystal can be attributed to its relatively high surface energy. Considering that the Au decoration occurs mostly on the larger area flat basal (100) facet from the experimental results, we have chosen the SnSe(100) surface for the characterization of the Au/SnSe nanocomposite.

**Table 1:** Comparison of turn-on value with metal chalcogenides and Au coated semiconducting nanostructures.

Number	Material	Morphology	Turn-on field (V/µm) for (10 µA/cm^2^)	Reference
1	Au/SnSe	Nano-heterostructure	1.25	Present work
2	SnSe	Nanosheet	2.25	
3	ZnS	Nanobelts or nanoribbon	3.55	^28^
4	ZnSe	Nanoribbons	5 (0.1 mA/cm^2^)	^29^
5	CuS	Nanowalls	8.5	^30^
6	ZnTe	Thin film	7.5	^31^
7	CdTe	Nanowires	2.2	^32^
8	Au/Si	Nanowires	1.95	^16^
9	Au/ZnO	Nanocomposite	2.65	^17^
10	Au/BN	Nanocomposite	3.9 (for J = 10 nA/cm^2^)	^33^
11	Au/TiO_2_	Nanocomposite	2.8	^18^

**Figure 6:**
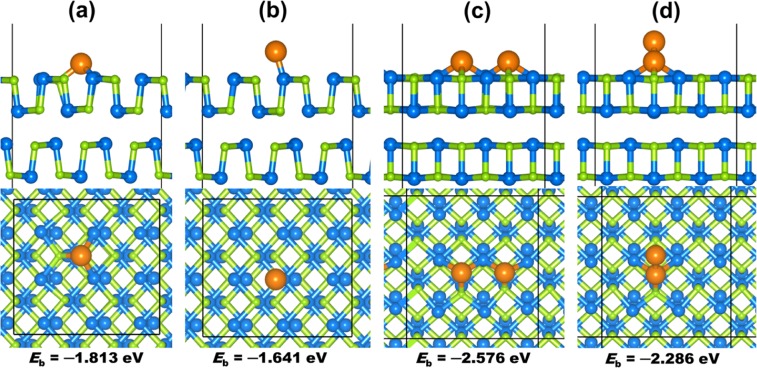
Optimized structures and average binding energies (${E}_{ab}$) of Au monomer ((**a**)-3fold-2Sn-Se and (**b**) top-Sn) and dimer ((**c**) 3fold-2Sn-Se and (**d**) Au-Au dimer) on SnSe (100) surface. Color scheme: Sn = blue Se = green, Au = orange.

To determine the lowest-energy structures of Au/SnSe (100), the average binding energy (${E}_{ab}$) of Au atoms was calculated as follows: ${E}_{ab}=1/n({E}_{Au/SnSe}-{E}_{SnSe}-n\ast {E}_{Au})$, where ${E}_{Au/SnSe}$ is the total energy of the Au-SnSe (100) system, ${E}_{SnSe}$ is the total energy of the naked SnSe (100) surface, ${E}_{Au}$ is the energy of the single Au atom, and $n$ is the number of Au atoms adsorbed. According to the above definition, a negative value of ${E}_{ab}$ indicates an exothermic and a favorable adsorption process. To search the most stable configuration, we considered five high symmetry adsorption sites (Fig. 4d), *i.e*., top-Sn (directly above Sn), top-Se (directly above Se), bridge-Sn-Se (bridge site between Sn and Se), 3fold-2Sn-Se (site that allows 2(Au–Sn) and Au–Se interactions), and 3fold-Sn-2Se (site that allows (Au–Sn) and 2(Au–Se) interactions). When a single Au atom at top-Se and bridge-Sn-Se sited it moved to the most stable 3fold-2Sn-Se site during energy minimization. When adsorbed at the 3fold-Sn-2Se site it moved to the top-Sn site, indication that only two stable Au/SnSe adsorption structures are possible as shown in Fig. 6(a,b). The most stable adsorption site for a single Au atom is predicted at the 3fold-2Sn-Se site (Fig. 6a), where the Au atoms form 3-fold coordination with two Sn and one Se atoms releasing a binding energy of −1.813 eV. The average Au–Sn and Au–Se bond distances are calculated at 2.740 and 2.564 Å, respectively. The binding energy for the top-Sn adsorption geometry (Fig. 6b) is calculated at −1.641 eV, with the Au–Sn distance converged at 2.592 Å. For the adsorption of two Au atoms, we have investigated the four different possible scenarios with the second Au atom binding on top of the preadsorbed Au to form a dimer (Au–Au), or binding at adjacent top-Se, bridge-Sn-Se or 3fold-2Sn-Se sites. When the second Au atom is adsorbed at adjacent top-Se site it moved to Au-Au geometry where the bridge-Sn-Se site structure converts to 3fold-2Sn-Se sites structure during energy minimization. The binding energy of the Au dimer (Au–Au = 2.564 Å) configuration (Fig. 6c) is calculated at −2.286 eV compared to −2.576 eV for the structure wherein both Au atoms bind at adjacent 3fold-2Sn-Se sites with 4.099 Å between them (Fig. 6c). This result illustrates that, at the 3fold-2Sn-Se site, the Au–Sn and Au–Se bonds are stronger than the Au–Au bond and the Au atoms initially tend to cover the 3fold-2Sn-Se site, indicating the selective adsorption at the initial growth stage with Au atoms covering the whole 3fold-2Sn-Se sites. That is to say that Au atoms prefer to wet the SnSe (100) surface rather than aggregate.

**Figure 7:**
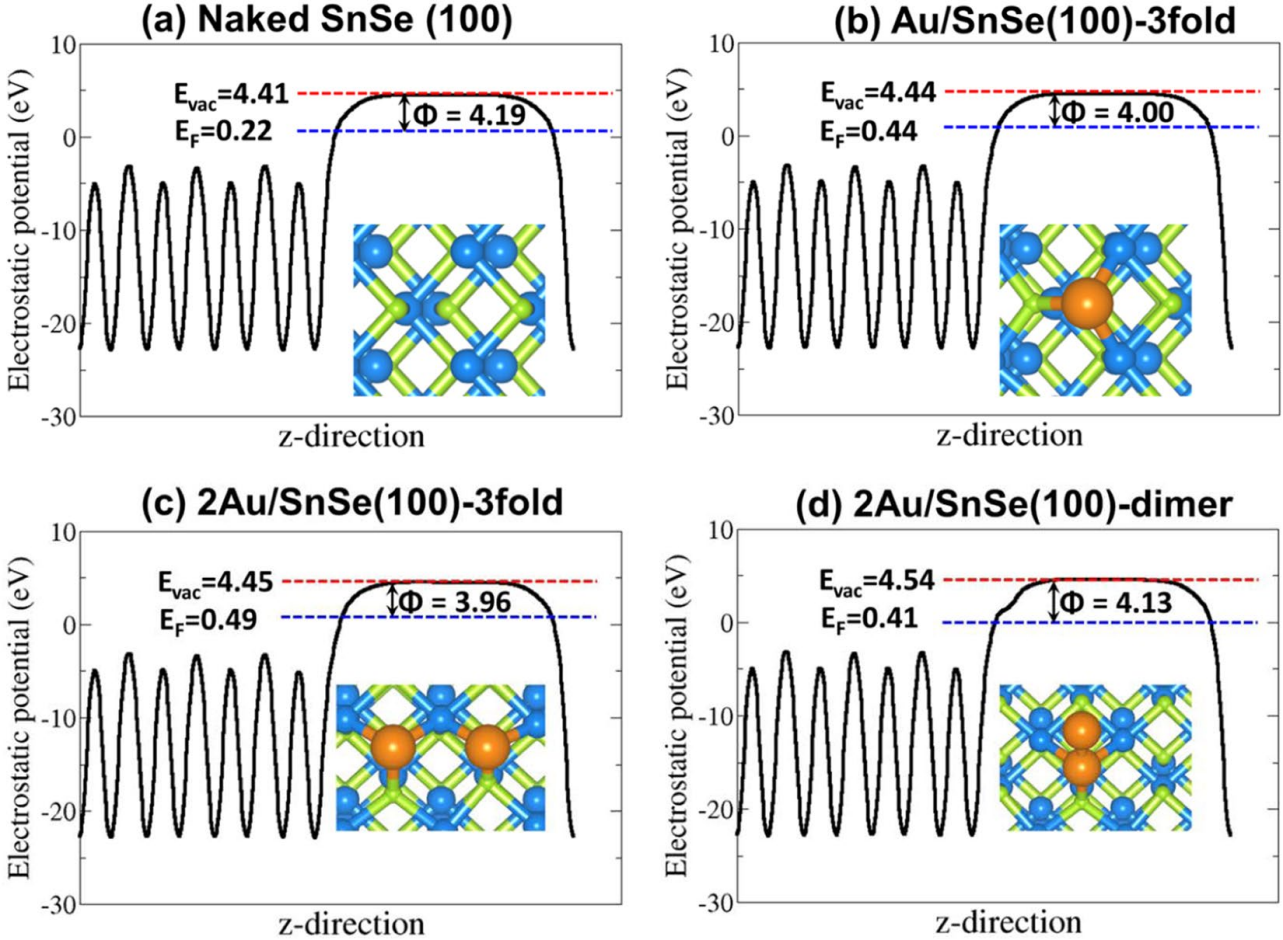
The electrostatic potentials for the (**a**) naked SnSe (100) surface, (**b**) the Au/SnSe (100) surface, (**c**) the 2Au/SnSe (100) surface with Au at 3fold-2Sn-Se site, and (**d**) the 2Au/SnSe (100) surface with Au-Au dimer configuration. The red and blue dashed lines represent the vacuum level (E_vac_) and the Fermi level (E_F_), respectively. The Φ is the work function.

Analysis of the work function for naked SnSe (100) and the Au-covered Au/SnSe (100) surfaces can help us to understand the origin of the enhanced field emission characteristics. The calculated work function of the naked SnSe (100) is 4.19 eV vs. vacuum as shown in Fig. 7a. The work function of the naked SnSe (100) surface is larger than that of the Au/SnSe (100) and 2Au/SnSe (100) surfaces as shown in Fig. 7(b–d). The work function of the most table Au/SnSe (100) structure (Fig. 6b) is 4.00 eV, that is 0.19 eV lower than the naked SnSe (100) surface. For the 2Au/SnSe (100) surfaces, the work function for the most stable adjacent 3fold-2Sn-Se (Fig. 7c) and Au-Au dimer (Fig. 7d) and configurations are 3.96 and 4.13 eV, respectively. It is also expected that the decoration of the edge (001) and (111) facets with Au nanoparticles would lead to a reduction of their work function, considering the fact that the adsorption acts to smoothen the surface electric charge distribution (the Smoluchowski effect) which generally results in lowering the work function^38,39^. As the field emission is favored for materials with lower work function, the predicted decrease in the work function of SnSe (100) surface with Au coating suggest the field emission can be greatly increased.

## Conclusion

Successful controlled synthesis of SnSe NSs and Au/SnSe NHS is demonstrated through one-pot colloidal and sputtering methods. XRD measurements confirm the formation of single-phase orthorhombic SnSe and FSEEM analysis shows dense nanoparticle coverage on the SnSe NSs, indicating formation of Au/SnSe NHS. By decorating the SnSe NSs with Au nanoparticles, significant improvements in field emission characteristics were observed. For instance, SnSe NSs and Au/SnSe NHS exhibited turn-on field of 2.25 and 1.25 V/µm respectively, which represents a 1 V/μm reduction in turn-on field. Besides, the field required to achieve high emission current density of 300 µA/cm^2^ is significantly reduced by 2.09 V/µm for Au/SnSe NHS. The I-t plot, which remain quite stable without showing any sign of diminishing over the 2 h period of continuous measurement also demonstrats the robustness of Au/SnSe NHS. Seeing that field emission is a surface sensitive phenomenon, a possible reason for the superior field emission characteristics of the Au/SnSe NHS compared to the isolated of SnSe NSs can be attributed to the surface modification of the SnSe NSs with dense Au nanoparticles. Being an electron source, the decoration of the SnSe NSs with Au nanoparticles helps to tune the electronic property of the Au/SnSe NHS towards the observed improved field emission characteristics and this is consistent with our earlier reports^18,19^. Also, the quasi aligned nature of SnSe NSs with narrow thickness and the decoration of the entire surface of SnSe NSs with very tiny Au particles (average diameter 15 nm) is expected to induce a high local electric field and hence the observed enhanced field emission properties. Besides that, the efficiency of a field emitter is strongly dependent on the emitter material’s work function, which dictates it’s the electron emission capability. In light of this, the predicted lower work functions from our first-principles DFT calculations for the Au/SnSe NHS (4.00 eV) compared to the isolated SnSe NSs (4.19 eV) is another possible origin for observed enhanced improved field emission characteristics of the Au/SnSe NHS compared to the isolated SnSe NSs. The investigations and analyses presented in this study did not only give a possible interpretation to the field emission characteristics of Au/SnSe NHS but also point out an efficient way of improving the field emission characteristics in the related nanostructures via surface and electronic modifications.

## Methods

### The preparation of SnSe NSs

**Figure 8:**
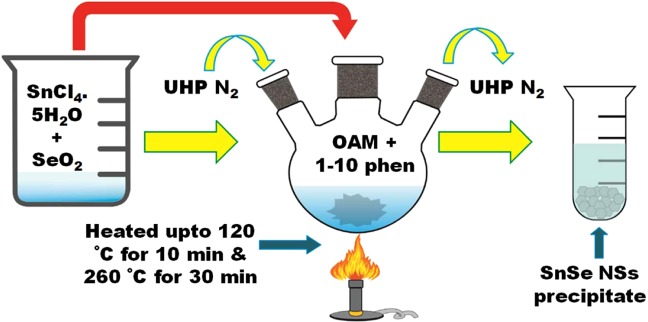
Schematic for the synthesis of SnSe NSs.

The synthesis of SnSe NSs is done by one-pot colloidal method. Shown in Fig. 8 is the schematic of the synthesis route of SnSe NSs. In brief, tin chloride pentahydrate (SnCl_4_:5H_2_O) 0.1 M, selenium dioxide (SeO_2_) 0.1 M, 1–10 phenanthroline (1–10 phen) 0.1 M and 10 ml of oleylamine (OAM) were added in a 3-neck flask. Here, the OAM acts as capping ligand as well as reductant. The mixture of chemicals in the 3-neck flask was stirred vigorously for 5 min and then degassed by Ultra High Pure (UHP) nitrogen (N_2_) gas. The solution was then heated at 130 °C and degassed by UHP N_2_ for 10 min. The mixture was again heated at 260 °C and aged for 30 min under UHP N_2_ condition. The black precipitated product formed at the bottom of the flask was purified in a centrifuge machine operated at 4500 rpm for 5 min through the addition of ethanol.

### The preparation of Au/SnSe NHS

**Figure 9:**
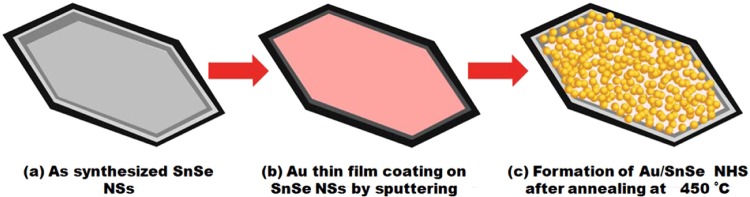
Schematic of the formation of Au/SnSe NHS.

A thermal annealing method^18^ was adopted for the synthesis of Au/SnSe NHS. In brief, a thin film of Au was coated onto the SnSe NSs for duration of 40 sec using Hitachi, E 1010 Ion sputter. The schematic representation of the Au deposition process on SnSe NSs to form the Au/SnSe NHS is given in Fig. 9. Further, annealing process of Au coated SnSe NSs is carried out at 450 °C for 1 h in air

### Materials characterization

X-ray diffraction studies were carried out by Rigaku (mini flex 600) for scan rate of 1 degree/min. A Field Emission Scanning Electron Microscope (FESEM Model - Hitachi S-4800) was used to examine the morphology and surface topography of the Au/SnSe NHS. Field emission measurements were carried out as stated in earlier report^40^. In present case, the area of both specimens (SnSe NSs and Au/SnSe NHS) was 0.24 cm^2^ and the separation between the anode and cathode was 1 mm. SnSe NSs and Au/SnSe NHS were directly pasted on highly conducting carbon tape.

### Computational methods

The first-principles DFT calculations were performed using the Vienna Ab initio Simulation Package (VASP)^41–43^, a periodic plane wave DFT code which includes the interactions between the core and valence electrons using the Project Augmented Wave (PAW) method^44^. An energy cut-off of 600 eV, and 3 × 7 × 7 Monkhorst-Pack *k*-point mesh^45^, was used to sample the Brillouin zone of bulk SnSe. All calculations were deemed to be converged when the forces on all atoms reached 0.001 eV/Å. The electronic exchange–correlation potential was calculated using the Perdew–Burke–Ernzerhof (PBE) generalized gradient approximation (GGA) functional^46^. For accurate determination of the electronic structure (partial density of states) the screened hybrid functional HSE06 (α = 0.25 and ω = 0.11 bohr^−1^)^47^ was used with a higher k-points mesh of 5 × 9 × 9. The bulk SnSe was modelled in the orthorhombic structure (Fig. 4) with space group (Pnma (No. 62)). From a full geometry relaxation, the lattice constants of SnSe were predicted at *a* = 11.571 Å, b = 4.168 Å and c = 4.517 Å, which are in good agreement with the experimental values (a = 11.550, b = 4.153, and c = 4.450 Å). The optimized of Sn–Se bond lengths were calculated to be 2.78 and 2.83 Å, which also agree well earlier theoretical prediction^48^. The SnSe (100), (001), (011), and (111) surfaces surface were created from the optimized bulk material using the METADISE code^49^, which ensures the creation of surfaces with zero dipole moment perpendicular to the surface plane^50^. Based on the calculated surface energies, the equilibrium Wulff morphology for the SnSe nanocrystal was constructed using the GDIS software^51^. In order to align the energies to the vacuum level, a slab-gap model (slab thickness of 20 Å and vacuum size of 15 Å) was constructed and the corresponding electrostatic potential was averaged along the *c*-direction, using the Macro Density package^52–54^, as displayed in (Fig. 7). The work function (Φ) of the naked SnSe(100) surface and the gold covered Au/SnSe(100) NHS was calculated as Φ = V_vacuum_ − E_F_ (where V_vacuum_ and E_F_ are the vacuum and Fermi level, respectively).

## Data Availability

Information on the data that underpins the results presented here, including how to access them, can be found in the Cardiff University data catalogue at http://doi.org/10.17035/d.2020.0098900038.
